# The great hairball gambit

**DOI:** 10.1371/journal.pgen.1008519

**Published:** 2019-11-26

**Authors:** Jonathan Flint, Trey Ideker

**Affiliations:** 1 Department of Psychiatry and Biobehavioral Sciences, University of California Los Angeles, Los Angeles, California, United States of America; 2 Division of Genetics, Department of Medicine, University of California San Diego, La Jolla, California, United States of America; The University of North Carolina at Chapel Hill, UNITED STATES

It’s generally agreed that the successful application of genome-wide association studies (GWAS) to the genetic dissection of psychiatric disease, resulting in the identification of hundreds of susceptibility loci for schizophrenia [1] and smaller numbers for depression [2, 3], autism [4], bipolar disorder [5], and anorexia [6], hasn’t been matched by equal success in working out the biology of the same conditions. To get at biological mechanism, one thing that defenders of the GWAS methodology generally propose and instantiate in their papers is a description of relevant molecular pathways and interaction networks–a.k.a network analysis–which takes as its starting point risk loci occurring at or close to genes.

The construction of gene and protein networks, whether made from correlated expression of transcripts or the interaction partners of proteins, can either be lauded as a way to transform information about genetic risk loci from genes to etiological mechanisms, or derided as an uninformative exercise, flawed not only by the poverty of the data upon which it relies, but more fundamentally by its departure from reductionist explanations of how things work in biology. The divide between these two positions sometimes seems to be as wide as the gulf between Republican and Democrat, or between the supporters and opponents of Brexit. Somewhere, presumably, there is a bridge across…

Network analysis has the potential to shed light on GWAS loci due to interactions at multiple layers of cell biological information. First, multilayered networks involving cooperative and antagonistic combinations of histone modifications, sites of transcription factor occupancy, enhancers, promoters and genes, create co-regulation of gene transcription. As a consequence, any genetic variant that disturbs the expression of a transcription factor or histone modifier typically has knock-on effects on the expression of many other genes, and even variants in genes that are supposedly regulated downstream of all this can cause a readjustment of transcription in the larger system. Second, protein interaction networks are central to cell and tissue biology, otherwise there’d be no organization of proteins into large multimeric complexes, or co-localization in specific subcellular compartments. Currently at least 4,400 mammalian proteins have been placed in specific protein complexes [7] and greater than two-thirds of proteins have been mapped to highly organized spatial compartments within the nucleus, cytosol, mitochondrion or membranes [8]. Without protein interactions there would also be no basis for signaling networks and the transmission of inter and intracellular signals, for instance from ligand to receptor or from kinase to substrate. Finally, metabolic networks provide a means by which changes in the levels or activities of enzymes or metabolites can propagate to affect the levels or activities of many others. In short, these many layers of networks attest to the fact that most genes don’t act in a vacuum, and thus to understand disease we need to know how individual effects alter larger biological processes modeled by networks.

All of these networks (transcriptional, structural, signaling, metabolic) are perturbed by variants at genetic loci underlying psychiatric disease. As with other complex traits, most of the risk loci for psychiatric disease lie in regulatory regions of the genome [9], which means that rather than altering protein structure they alter mRNA production or mRNA stability via transcriptional networks. In turn, alterations to transcription affect protein networks, through changes in the stoichiometry of protein complexes and the activities of signaling cascades. They affect metabolic networks through changes in enzyme abundance, which then give rise to many indirect effects on metabolic levels and fluxes more globally. Ultimately, the collection of all network alterations in an individual translates to a distinct profile of effects on psychiatric development, behavior and therapeutic outcomes. Charting causal pathways through these networks, i.e. connecting causal genetic variants to impacts on transcriptional, protein, and/or metabolic networks to impacts on psychiatric phenotypes, is the essence of network analysis.

All this sounds very convincing, but to what extent has network analysis thrown new light on disease pathogenesis? There’s certainly evidence that gene expression analysis identifies expression modules that contain GWAS signals, for example from autism spectrum disorder [10] and schizophrenia [11]. What’s less certain is the utility of this information. You’ll read that network analysis has indicated that autism and schizophrenia are disorders of the synapse [12, 13], and that autism is characterized by disorders of cortical projection neurons from midfetal layer 5/6 [14]. That doesn’t, yet, amount to a mechanistic interpretation, other than in a broad sense. What we really want to know when we ask about the biological causes of psychosis is *what is wrong with the synapse*, *and how does that explain psychiatric phenomena*?

Perhaps a lack of detailed information on where and when in the brain genes are expressed limits the power of networks to identify causal mechanisms, a view that drives the collection of ever larger datasets, for instance taken from single cells at different developmental time points [15]. In the meantime, we can think of several major reasons for why networks may fall short of delivering the causal pathway explanations everyone is looking for.

First, the starting point for network analysis, a set of genes associated with a disease, does not fall out of the data like factors from a principal component analysis. Translation of genetic loci to genes is more often a matter of faith than of rigorous proof. Despite the presumptions of many bioinformatics programs (e.g. ALIGATOR, INRICH MAGENTA and MAGMA [16–20]), proximity to a GWAS signal is not a guarantee for gene identification. As a series of experiments have shown, regulatory elements containing SNPs don’t always regulate the nearest gene [21–23]. Rather, the vast majority of GWAS loci are in regulatory regions that lie at a distance, sometimes many megabases, from their gene targets [9]. One estimate is that about half the targets are in fact the closest gene [24] whereas, roughly speaking, about half are incorrect when using genomic proximity as the sole indicator. Rigorous demonstration that the causative variant at a risk locus has been identified, the effect of that change confirmed, and the target gene found has been slow coming in the GWAS field, largely because it is such a difficult thing to do [21].

Second, even if we had accurate sets of genes from GWAS, we are still far from having complete interaction maps. Arguably, resources such as BioPlex [25] and the yeast-two-hybrid (Y2H) Human Reference Interactome [26] are nearing complete coverage of the human protein-protein interaction space, but these are either in one cell line (BioPlex is in HEK293T cells, workhorses of protein production but of unclear relevance for neurobiology) or, in the case of yeast two hybrid, not in a human cell type. Then there is the question of biological context, including the relevant growth conditions, tissue microenvironment, state of functional activation (especially relevant for the brain), and course of therapy. Even in a single biological context, network mapping technologies have typically large false negative rates [27] meaning that many true interactions are missed, and methods that query interactions with a single partner introduce biases that at least have the virtue of being acknowledged.

As with protein network mapping, attempts at completeness of transcriptional profiling data are vitiated by the temporal, tissue and cell specific nature of what gets expressed. Except in constrained situations of analysing a single cell type, at the same point in the cell cycle, in unvarying environmental situations, with multiple measures taken over a uniform timeframe, we cannot obtain fully complete or reliable measures of transcriptional state. Furthermore, our dependence on what is most studied, such as cancer cells, contaminates annotations, and one can only wonder at what biases remain to be detected.

A third fundamental problem facing networks is their lack of specificity. In co-expression networks everything correlates with everything at some level, including both upstream causes and the multiple consequent downstream effects. The result is an enormous hairball in which, to a first approximation, every gene interacts with every other gene. Issues with specificity beset other network types too. For instance, protein interaction screens identify proteins by their affinity to a tagged “bait” which is typically overexpressed at dramatic levels. The degree to which such overexpression leads to specificity problems is not well characterized. Surely these specificity problems increase the difficulty of identifying a pathway causal for a disease?

A final, and implacable, enemy of network analysis is the genetic architecture of psychiatric disorders. Ever since David Goldstein plotted the distribution of effect sizes of loci for complex traits [28], it’s been a source of worry to geneticists that explaining heritability is going to require a very large number of loci. Just how many still isn’t known, but it’s likely to be tens of thousands. For example, greater than 100,000 SNPs have been identified with independent effects on human height, perhaps extending upwards to as many as half of common SNPs [29]. There’s every reason to believe that psychiatric disorders are similarly polygenic. In fact, one need hardly extrapolate to predict that the number of genes associated with such disorders will soon saturate at close to all 20,000 genes. Then what? What more will we really understand about the diseased genome, not to mention its molecular mechanisms or resulting physiology?

None of this is to say that biological explanations eschew networks, but how can we advance molecular network maps along a productive path for doing human genetics? First off, at least in their present form co-expression data may simply be the wrong type of network information for the job of reading the genome. No matter how complete they are, they tell you very little about mechanism, since they are dominated by knock-on effects and massive gene-expression regulons. The causal change in expression, the one that can illuminate the pathway from gene to disease, is lost in a haystack of thousands of other changes, or, worse still, the causal pathway is just simply unobservable at the level of gene expression. Furthermore, simply computing correlations in gene expression over all available samples, as is often the practice, is confounded by issues of physiological context and cell type–what may be really going on is that the ‘co-expressed’ genes are specifically active in one cell type, the frequency of which varies across experimental subjects.

Co-expression networks are widely used because they are conveniently generated from high-throughput sequencing, not because they are necessarily the right structure for genetic interpretation. Strikingly, in a systematic assembly of autophagy pathways [30] mRNA co-expression could be dispensed with *entirely* without loss of information, whereas other network types such as protein-protein and synthetic-lethal interactions were critical for reconstructing current knowledge of pathway relationships and functions. Will the same conclusions hold when different network data types are evaluated for their power in interpreting psychiatric GWAS? Thus far it appears that networks integrating multiple types of evidence are significantly more useful for interrelating disease loci than those representing one data set or type only [31]—good to know but hardly surprising.

Alongside network type are considerations of biological conditions. Networks should be mapped in the cellular and environmental conditions most relevant to psychiatric disease, while continually working in contexts in which systematic datasets can be readily generated. Whether this means selecting a suitable panel of neuronal cell lines or organoids, or going straight to brain or other tissues, remains to be determined. Moreover, in making these decisions we need more information about the stability of transcriptional, protein, and metabolic networks to genetic and environmental perturbations, as well as to experimental errors. At one extreme, network architecture might be so ephemeral as to require separate maps in each individual, or worse yet, every clonal cell population within each individual. At the other extreme, perhaps one or a few reference networks are enough to understand the etiology of most cases of a disease. Regardless, having *an initial* draft set of network reference maps for neuropsychiatric disease would represent a big leap forward from where we are now, as has recently been undertaken by consortia like SynGO [32].

Given that only a dozen years ago almost nothing was known about the genetic loci underlying behavior, the success of GWAS is remarkable: 145 loci for schizophrenia [1], 102 for depression [3], 202 for insomnia [33] and 1,271 for educational attainment [34]. Similar troves of loci are accumulating for most other complex genetic diseases, including cancer, cardiovascular disorders, diabetes, and rheumatoid arthritis. Access to large biobanks [35] together with cheap genotyping and data sharing has made it relatively straightforward to find loci. Perhaps that very success has encouraged the view that the functional interpretation of genetic signals has become equally routine.

On the contrary, our opinion is that the difficulties of integrating network and genetic data are under appreciated. The risk is that many of the networks currently reported to be responsible for psychiatric disease will turn out to be the equivalent of the false-positive findings that emerged from the early genetic studies of behavior: incomplete results, obtained from the wrong type of data in the wrong cellular conditions, using the wrong genes, conspire to create hairballs of questionable value. Before we can obtain robust functional interpretations of GWAS findings, we’d like to see a comprehensive collection of network and pathway datasets for psychiatric and other complex disorders, boosting network coverage and accuracy, and integrating data from multiple sources, rather than the common practice of relying solely on expression data.
